# Sinonasal Undifferentiated Carcinoma (SNUC): the Alberta experience and literature review

**DOI:** 10.1186/1916-0216-42-2

**Published:** 2013-01-31

**Authors:** Caroline C Xu, Peter T Dziegielewski, William T McGaw, Hadi Seikaly

**Affiliations:** 1 Department of Surgery, Division of Otolaryngology-Head and Neck Surgery, University of Alberta, 8440 112 Street NW, Edmonton, Alberta, T6G 2P4, Canada; 2 Department of Dentistry and Dental Hygiene, University of Alberta, 8440 112 Street NW, Edmonton, Alberta, T6G 2P4, Canada; 3 Department of Laboratory Medicine and Pathology, University of Alberta, 8440 112 Street NW, Edmonton, Alberta, T6G 2P4, Canada

## Abstract

**Background:**

Sinonasal undifferentiated carcinoma (SNUC) is a rare malignancy with often dismal outcomes. This study set to determine provincial and literature-wide survival outcomes based on treatment modality.

**Methods:**

Retrospective chart review of all SNUC patients in the province of Alberta from 1986–2010 was conducted. A review of the literature of SNUC patients was also performed. Patient/tumor characteristics, treatment, and follow-up/survival data were collected. Kaplan-Meier and Cox regression survival analyses were performed.

**Results:**

20 patients were treated for SNUC in Alberta and 140 patients were identified in the literature. Pooled median disease-free survival was 12. 7 months and 5-year survival estimate was 6.25%. Cox-Regression analysis demonstrated an overall survival advantage with multimodality treatments (Log-Rank test: p = 0.015). However, no statistically significant differences in disease-free and overall survival were identified between patients treated with chemoradiation or surgery followed by adjuvant therapy.

**Conclusions:**

Treatment of SNUC remains challenging with poor survival outcomes. There appears to be no statistically significant difference in overall, or disease-free survival between treatment modalities.

## Introduction

Sinonasal undifferentiated carcinoma (SNUC) is a rare, highly aggressive and clinico-pathologically distinctive carcinoma of uncertain histogenesis [1]. The disease affects males more often than females and has a broad age range [2]. SNUC presents as a rapidly enlarging tumour arising from the sinonasal tract with initially vague symptoms [3] that are of relatively short duration. Orbital, dural, and intracranial invasion are common [2,4] at presentation.

Pathological examination of SNUC typically reveals large tumours with fungating and poorly defined margins that invade adjacent structures [1]. The histologic appearance is characterized by sheets, trabecular, and ribbon-like arrangements of small to medium-size undifferentiated cells. These cells often have high nuclear to cytoplasmic ratio, high mitotic rate, and prominent tumour necrosis [3]. Lymphovascular and neural invasion are often also identified [3]. Immuno-histochemical antigenic profiles vary widely [3], however, features of neuroectodermal differentiation are typically absent [5].

The majority of patients present with advanced stage disease and often undergo intense, multi-modality treatment [6]. Unfortunately, survival remains poor [1,2,7,8]. The purpose of this study was to evaluate survival outcomes based on treatment of SNUC patients in Alberta and the literature at large.

## Methods

Ethics approval was granted by the University of Alberta *Health Research Ethics Board* (HREB) committee as well as the Alberta Cancer Board. The study was conducted at a tertiary care academic referral centre.

### Provincial chart review

A systematic, retrospective medical record review was performed. All patients diagnosed with SNUC in the province of Alberta from 1986–2010 were identified in the Alberta Cancer Registry [9], which is a certified member of the North American Association for Central Cancer Registries. Charts and electronic medical records for all identified patients were then accessed and cross-referenced to confirm suspected diagnoses of SNUC. Tumors of all patients were then pulled from a tumor bank and analyzed by a single head and neck pathologist (WTM). Any tumors with questionable pathology reports were also obtained and analyzed. The following criteria were then applied to these patients: 

Inclusion Criteria:

1) Histological diagnosis of SNUC

2) Treatment within Alberta

*Exclusion Criteria*:

1) Non-SNUC tumor

2) Incomplete data

Medical records were then gleaned for: patient, tumor and treatment characteristics as well as follow-up time, survival status, and disease-free status as of March 30^th^, 2010. Patient age was recorded as the age on the first day of primary treatment. Clinical TNM staging was obtained from multi-disciplinary tumor board notes, which followed the American Joint Committee on Cancer guidelines [10]. Overall survival was defined as the time from the first day of treatment to death. Disease-free survival was defined as the first day of treatment to disease recurrence.

### Literature review

A literature review was undertaken in a systematic fashion using: MEDLINE, EMBASE, Pubmed, and Scopus. The goal was to identify papers describing the survival of SNUC as per treatment modality. Three reviewers searched for the following terms: “sinonasal undifferentiated carcinoma”, “SNUC”, “undifferentiated carcinoma”, and “paranasal sinuses”. Foreign language studies were included in the search strategy and only excluded from the review if an English translation was not available. Bibliographies were also checked for relevant publications. Abstracts of publications were screened for possible inclusion (as per above criteria) and relevant articles were reviewed in full. Studies were excluded if survival time and status at last follow-up were not provided. Each study was evaluated for gender and age of the patient in the study, presenting symptoms, orbital involvement, treatment strategy, and follow-up. Where AJCC staging was not provided, the Kadish staging was converted to an AJCC TMN stage based on information provided.

### Statistical analysis

Actuarial survival for pooled patients was estimated via Kaplan-Meier analysis to account for censored data. A log rank test was used to compare survival curves with statistical significance set as p<0.05. Cox-regression analysis was also performed. Covariate factors known to influence survival including age, gender and tumor stage were incorporated as potential confounding variables. Statistical analysis was conducted using SPSS 19.0 (SPSS Inc., Chicago, IL).

## Results

20 histologically confirmed cases of SNUC were identified in Alberta. The majority of patients were male, middle-aged, and presenting with Stage IV disease (Table 1). Patients presented with headache or neurologic symptoms (45%), nasal obstruction (40%), epistaxis (25%) and occasionally, facial pain (5%). In many patients, the exact tumour origin within the sinonasal tract was difficult to determine due to the extensive nature of their tumours on pre-operative imaging.

**Table 1:** Baseline variables of SNUC patients in Alberta

**Variable**		**Total Number (%)**
**Patients**		20
**Males**		16 (80)
**Females**		4 (20)
**Mean age at diagnosis, years (range)**		53.9 (35–85)
**Mean follow-up time, months**		21
**Stage at presentation**	**Stage IV**	17 (85)
	**Stage III**	2 (10)
	**Stage II**	1 (5)
**Tumor origin site**	**Sinonasal – not otherwise specified**	7 (35)
	**Nasal cavity**	4 (20)
	**Maxillary sinus**	4 (20)
	**Ethmoid sinus**	5 (25)

### Systematic review and pooled analysis

42 potential articles were identified through a systematic and comprehensive search. 26 articles met study criteria and yielded 140 patients available for survival analysis (Table 2). Patients in the literature presented with symptoms of nasal obstruction (20.0%), epistaxis (17.1%), diplopia or other visual symptoms (15.0%), or headache (12.1%). Rarely, patients also presented with facial pain (7.1%) or sinusitis (2.9%). At presentation, 42.9% of patients had orbital involvement. AJCC TNM staging was available for 128 (87.6%) of the patients with the majority comprising of Stage IV disease (Table 2).

**Table 2:** Patient demographics of SNUC patients in the literature

**Variables**		**Number of patients (%)**
**Patients**		140
**Males**		107 (76.4)
**Females**		33 (23.6)
**Mean age, years (range)**		52.5 (8-84)
**TNM Stage**	**Stage IV**	102 (72.9)
	**Stage III**	15 (10.7)
	**Stage II**	11 (7.9)
	**Not Stated**	12 (12.4)
**Mean follow-up time, months**		22

The majority of patients in the literature (66.4%) received multimodality therapy (Table 3). Where recurrence data was available (107 patients), the recurrence rate was 42.3% with time to recurrence ranging from 3–33 months. 32.1% of patients died of local disease while 14.3% of patients died of metastatic disease. The most common sites of metastasis were bone (10.2%), cervical lymph node (9.5%), lung (5.8%), brain (5.8%), and liver (4.4%). The pooled median disease-free survival was 12.7 months. The 1, 3 and 5-year survival estimates were 51.2%, 19.4% and 6.25% respectively. The median overall survival was 12.7 months.

**Table 3:** Treatments received by SNUC patients

** Modality**	**Number of patients (%)**	
	**Alberta**	**Literature**
**No treatment**	2 (10)	5 (4)
**Single modality**	7 (35)	42 (30)
**Chemoradiation**	4 (20)	37 (26)
**Surgery + Adjuvant radiotherapy**	3 (15)	22 (16)
**Surgery + Chemoradiation**	4 (20)	34 (24)

Only two patients in Alberta received no treatment (Table 3): one died days after presenting with a brain abscess and another patient opted for no treatment. The remainder of patients received either a single or multi-modality approach. In nine of the 20 patients, metastasis was detected within an average of 12.7 months (standard deviation of 33.2 months, range: 2.6 months to 3.7 years). The mean time from recurrence to death was 4.9 months (standard deviation of 3.2 months, range: 1.0 to 7.8 months). Seventeen patients (85%) died of their disease while the remaining patients died of respiratory failure and/or aspiration pneumonia.

**Figure 1:**
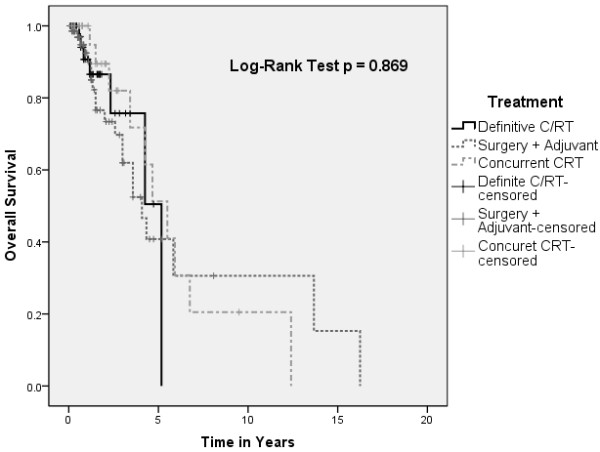
Kaplan-Meir plot depicting overall survival by treatment modality.

**Figure 2:**
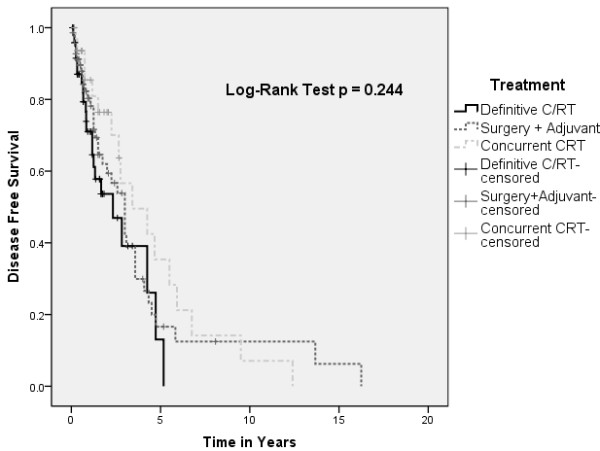
Kaplan-Meir plot depicting disease-free survival by treatment modality.

**Figure 3:**
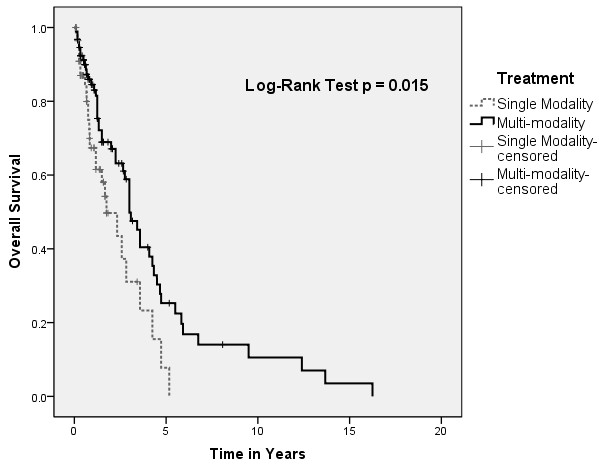
Kaplan-Meir plot depicting overall survival by single versus multi-modality approaches.

**Figure 4:**
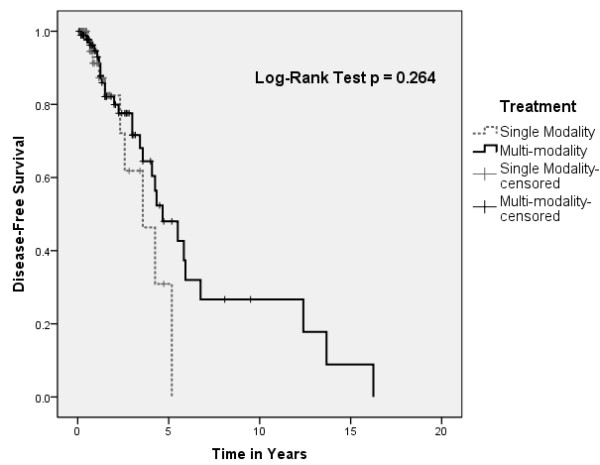
Kaplan-Meir plot depicting disease-free survival by single versus multi-modality approaches.

A statistical difference in overall survival of pooled patients was found between patients treated with single versus multi-modality approach (Figure 1). However, this did not translate to a difference in disease-free survival (Figure 2). There were no differences in overall and disease-free survival based on initial treatment, with Cox regression analysis to account for covariables such as age, sex, and stage (Figures 3 and 4).

## Discussion

Rare sinonasal tumours are challenging clinical entities for head and neck oncology and skull base surgeons [11-13]. Sinonasal undifferentiated carcinoma is a particularly difficult to treat.

We described the results of a series of 20 patients with SNUC treated between 1986 and 2010 in Alberta. As consistent with previous studies [4,14-19], the majority of these patients presented with locally advanced disease. At the senior author’s institution, patients with resectable tumours are offered surgery followed by chemoradiation. Chemotherapy agents used included carboplatin, cisplatin, and etoposide. Surgical resection typically involved extensive craniofacial resection with maxillectomy, orbital exenteration, and occasionally, neurosurgical involvement. Overall, outcomes of these patients were typical of those depicted in the literature: patients had high rates of recurrence, metastatic disease, and had very limited disease-free survival.

Kramer et al. [15] presented a case series and systematic review of 60 patients with SNUC in the literature in 2004. More recently, Reiersen et al. also presented a systematic review of patients in the literature [20]. Their study had similar findings of overall poor prognosis. However, the study authors proposed that treatment of SNUC patients should include surgery with adjuvant therapy. This is based on findings that surgery was the best initial modality and the addition of chemotherapy and radiation provided additional survival benefits. Our results also suggest that a multi-modality approach confers an overall survival advantage. However, our results failed to show a significant advantage of surgical-based treatment stategies and there appears to be no clear disease-free survival advantage with any particular modality, based on our results.

Our study has several limitations. First, this is a review and pooled analysis of an area of the literature comprised entirely of small to medium-sized case series. The data is heterogeneous and the quality is often poor. AJCC staging information was not available for all patients and often the Kadish staging system was necessarily converted to an AJCC stage based on available information regarding anatomic involvement. Firm conclusions are difficult to make due to the lack of statistical power, reporting bias, and heterogeneity of clinical characteristics and treatment modalities. However, this study confirms that despite advances in chemotherapy and radiotherapy, the cure-rate for SNUC remains dismally low. Recurrence rate is very high and many patients died of disease within months of diagnosis.

Given the challenges and limited success in the treatment of SNUC, alternative therapeutic modalities are clearly necessary. Novel therapies that have been attempted include boron neutron capture therapy [21], autologous bone marrow transplant [22], and neoadjuvant selective intra-arterial cisplatin with concurrent radiation therapy [23], both of which showed limited success. A recent histo-biochemical study of a small series of SNUC patients showed that these tumours have a strong expression c-KIT, which is a tyrosine kinase receptor typically expressed in gastrointestinal stromal tumours [24]. However, over-expression was not due to an activating mutation in the *c-kit* gene, and thus, the authors argued that use of TK-inhibitors targeting c-KIT (e.g. Imatinib/Gleevac) may not prove successful. However, this represents a possible new avenue of therapy.

## Conclusions

SNUC is a unique clinico-pathologic entity that remains a challenge to treat despite aggressive multidisciplinary approaches. There is no evidence that aggressive surgery and post-operative RT offer any survival advantage compared to other modalities. Therefore, a search for alternative therapies is warranted.

## Competing interests

The authors declare that they have competing interests.

## Authors’ contribution

CCX, PTD and HS designed study. CCX collected and analyzed data completed. WTM reviewed pathology specimens. CCX wrote the manuscript. All authors read and approved the final manuscript.
